# Acute kidney injury in non-critical care setting: elaboration and validation of an in-hospital death prognosis score

**DOI:** 10.1186/s12882-019-1610-9

**Published:** 2019-11-21

**Authors:** Jamal Bamoulid, Hélène Philippot, Amir Kazory, Maria Yannaraki, Thomas Crepin, Bérengère Vivet, Nadège Devillard, Caroline Roubiou, Catherine Bresson-Vautrin, Jean-Marc Chalopin, Cécile Courivaud, Didier Ducloux

**Affiliations:** 1 CHU Besançon, Department of Nephrology, Dialysis, and Renal Transplantation, F-25030 Besançon, France; 2 UMR1098, Federation hospitalo-universitaire INCREASE, F-25020 Besançon, France; 3 Faculté de Médecine et de Pharmacie, Université de Franche-Comté, F-25020 Besançon, France; 4 Structure Fédérative de Recherche, SFR FED4234, F-25000 Besançon, France; 5 Department of Medicine, University of Florida, Gainesville, Florida USA

**Keywords:** Cute kidney injury, In-hospital death, Prognosis score, Iatrogenesis, Score validation

## Abstract

**Background:**

Acute kidney injury (AKI) is still characterized by a high mortality rate. While most patients with AKI are admitted in conventional medical units, current available data are still obtained from studies designed for patients admitted in intensive care units (ICU). Our study aimed to elaborate and validate an in-hospital death prognosis score for AKI admitted in conventional medical care units.

**Methods:**

We included two prospective cohorts of consecutive patients with AKI admitted between 2001 and 2004 (elaboration cohort (EC)) and between 2010 and 2014 (validation cohort (VC)). We developed a scoring system from clinical and biological parameters recorded at admission from the EC to predict in-hospital mortality. This score was then tested for validation in the VC.

**Results:**

Three-hundred and twenty-three and 534 patients were included in the EC and VC cohorts, respectively. The proportion of in-hospital death were 15.5% (EC) and 8.9% (VC), mainly due to sepsis. The parameters independently associated with the in-hospital death in the EC were Glasgow score, oxygen requirement, fluid overload, blood diastolic pressure, multiple myeloma and prothrombin time*.* The in-hospital death prognosis score AUC was 0.845 +/− 0.297 (*p* < 0.001) after validation in the VC.

**Conclusions:**

Our in-hospital death prognosis score is the first to be prospectively developed and validated for AKI admitted in a conventional medical care unit. Based on current parameters, easily collected at time of admission, this score could be a useful tool for physicians and nephrologists to determine the in-hospital death prognosis of this AKI population.

## Background

Acute kidney injury (AKI) is defined as deterioration of renal function over a short period [1]. This definition has been formalized for optimal data comparison between clinical studies thanks to the 2004 RIFLE [2], 2007 AKIN [3] and 2012 KDIGO [4] classifications. These classifications determine several AKI stages based on either an increase in creatinine or decrease in glomerular filtration rate (GFR) and a decrease in urine output. Moreover, these stages help to stratify the severity of AKI and the risk of in-hospital death [5].

Based on these definitions, epidemiological studies reported incidences of 24.4 and 384.1 per 100,000 inhabitants per year, respectively in dialysis and non-dialysis requiring AKI [6]. A recent meta-analysis including 154 studies based on AKI KDIGO definition reported that 20% of hospitalized adult patients experience AKI whatever the initial cause of hospitalization [7]. Additionally, the incidence of AKI continuously increases from about 11% per year, particularly in male and elderly populations [8].

Despite several advances in therapeutics and substantial progress in the understanding of AKI pathogenesis, the related mortality remains high [9]. Overall in-hospital mortality is about 23% in adults [7]. In the setting of intensive care units (ICU), AKI-associated mortality ranges from 45 to 73.5% [10–12]. Interestingly, the etiology of AKI can influence mortality rate; epidemiological studies have shown that AKI secondary to nephrotoxic agents and drugs contributes to death in up to 30% of cases, which remains high but significantly lower than in other potential etiologies [13–15]. Moreover, AKI is a risk factor contributing to chronic kidney disease (CKD): the annual incidence of chronic dialysis is about 8.6% after one episode of AKI [16].

Most studies about AKI have been conducted in ICU. These studies have reported sepsis, as well as pulmonary, cardiac, and hepatic failure as independent risk factors for AKI-associated mortality [17–24]. Yet, few epidemiological data are currently available on patients admitted to conventional medical units for AKI. Indeed, among the 154 studies included in the recent meta-analysis of Susantitaphong et al*,* only 7 included patients from conventional hospitalization units of nephrology [7]. Many aspects of AKI, including etiologies and prognosis, might be different in this group of patients compared to that developing AKI in the ICU setting. Moreover, RIFLE, AKIN or KDIGO classifications are particularly difficult to use in everyday practice among conventional units, particularly the close surveillance of urine output.

In this study, we explored epidemiological and biological characteristics of patients admitted to a conventional medical service for AKI and assessed predictive factors for in-hospital death. Our study aimed to elaborate and validate an in-hospital death prognosis score for AKI admitted in conventional medical care units.

## Methods

### Patients

All patients admitted for AKI to the nephrology unit of the University Hospital of Besançon between January 2001 and December 2004 (score elaboration cohort (EC)) and then between January 2010 and December 2013 (score validation cohort (VC)) were prospectively considered for inclusion.

This study was conducted according to the code of public health and the code of medical ethics (6th January 1978 amended in 2004) concerning research that does not involve the human person (study based on health data); the General Data Protection Regulation 2016–679 on the protection of personal data (GDPR) (27th April 2016) and in particular Article 6.1.a highlighting the principle of consent to the processing of personal data; the law 78–17 (6th January 1978) relating to computer, files and freedoms, modified the so-called IT Law and Freedoms (LIL) and the Reference Methodology MR-004 of the French National Agency regulating Data Protection (CNIL). The number of the declaration of conformity to the reference methodology MR-004, concerning research not involving the human person, studies and evaluations in the field of health, is 2,214,506 v0 of July 24th, 2019. All patients participating in this study received written informed information and the opportunity to oppose the use of their data.

All patients were ≥ 18 years old at the admission date. AKI was defined either by an increase of 50% or more in serum creatinine (SCr) in patients with previous normal renal function, or a rapid increase ≥100 μmol/l in SCr for patients with previous chronic kidney disease (CKD) (*i.e* estimated GFR < 60 mL/min/1.73 m2 according to MDRD (Modification of the Diet in Renal Disease) over a period of 12 weeks. If no information was available on previous renal function before admission, clinical circumstances as well as reversibility of renal dysfunction and kidney size (evaluated by ultrasound) were considered to determine the acute nature of renal failure. Patients first admitted to ICU and then transferred to our unit and renal transplant recipients admitted for AKI were excluded.

### Methods

Relevant data were collected at the admission. These included demographic characteristics (gender, age, weight, height), provenance (home, rehabilitation center, nursing home, emergency, other hospital services), clinical setting (history of diabetes, hypertension, congestive heart failure, cirrhosis, cancer), systolic and diastolic blood pressure, fluid overload, oxygen therapy requirement, Glasgow coma scale, urine output (normal, marginal, oligo-anuria), and laboratory data (serum urea, Creatinine, Sodium, Potassium, Bicarbonate, Calcium, Phosphorus, Albumin, C-reactive protein [CRP], Hemoglobin, white blood cell count, coagulation parameters, and urinary protein excretion). Normal urine output was defined as a daily urinary volume equal or above 1000 ml; marginal urine output was defined as a daily urinary volume of 400–1000 ml and oligoanuria was defined as a urinary volume below 400 ml per day. Chronic kidney disease was defined as a GFR constantly below 60 ml/min/1.73m^2^ (estimated by MDRD) in the 6 months’ period preceding the admission. Etiology (when known), secondary diagnosis, dialysis requirement, renal biopsy, hospitalization duration, and outcomes (secondary transfer, death) were also recorded.

### Statistical analysis

The results were expressed as mean +/− standard deviation. The median and range were given for variables which distribution was not normal. We used a logistic regression model. First, univariate analyses were carried out to examine the relationship between death and several potential independent variables. Among the latter, covariates to enter multivariate analyses were selected as follows:

- Continuous variables were tested using Student’s t test or Mann-Whitney’s non-parametric test.

- Categorical variables were tested using Pearson’s chi-square, or Fisher’s exact test.

The alpha threshold for covariate selection was 0.20. These death-related variables with *p*-values < 0.20 were entered in the multivariate analysis model, medical knowledge of the variables guiding the final choice. Selected covariates were entered the stepwise multiple-regression analysis. Starting with many covariates as were chosen (either statistically or otherwise), the software performed backwards stepwise selection, the limit to remove a covariate being (p-value) 0.10.

An individual prognosis score was then built from the parameters independently associated with intra-hospital death in the EC cohort. The score discrimination was tested twice using ROC curve and Hosmer & Lemeshow test (a *p* value > 0.05 was considered statistically significant and associated with a good predictability of the score). In a second step, the score was validated in the VC cohort using ROC curve and Hosmer & Lemeshow test.

## Results

### Population characteristics

The cohorts’ characteristics are summarized in Table 1. Twenty-six patients out of 349 without complete data collection at the end of the study were excluded from the EC cohort, and only 323 patients were retained for analysis. There were no missing variables in the VC. Hence 323 and 534 patients were included in the EC and VC cohorts, respectively. The cohorts were comparable for age and about one-half patients were above 75 y-o. Two-third patients were male with about one-half CKD patients. The provenance was majoritarian from emergency room (48% (EC) vs. 57% (VC); *p* = 0.16), followed by other hospital units (31% vs. 34%; *p* = 0.71). Direct admissions (home) represented 16% (EC) and 7% (VC) of all admissions (*p* = 0.05).

**Table 1:** Elaboration (EC) and Validation (VC) cohorts’ characteristics

	EC (*n* = 323) VC (*n* = 534)		
	n (%)	n (%)	p ^a^
Male gender n. (%)	203 (63)	342 (64)	0.86
Mean age [min – max]	71.9 [19–99]	71.3 [18–99]	0.92
<50	27 (8)	53 (10)	0.69
50–75	135 (42)	208 (38.5)	0.63
>75	161 (50)	273 (52)	0.8
CKD^b^	144 (45)	285 (53)	0.21
Unknown previous renal status	0 (0)	13 (2)	0.12
Provenance			
Home	53 (16)	39 (7)	0.05
Emergency	154 (48)	306 (57)	0.16
Other hospital unit	101 (31)	181 (34)	0.71
Rehabilitation center	15 (5)	8 (1)	0.18
Etiologies			
Functional	149 (46)	208 (39)	0.3
Dehydratation	46 (14)	110 (21)	0.23
Drugs	34 (11)	8 (2)	0.007
Both	69 (21)	90 (17)	0.41
Obstructive	31 (10)	35 (7)	0.42
Glomerular disease	22 (7)	74 (14)	0.09
Acute tubular necrosis	51 (16)	116 (22)	0.28
Post-ischemia	44 (14)	66 (12)	0.78
Nephrotoxic agents	1 (0.3)	31 (6)	0.02
Rhabdomyolysis	6 (2)	19 (4)	0.46
Vascular disease	5 (2)	23 (4)	0.25
Multiple myeloma	21 (7)	12 (2)	0.13
Cardio-renal syndrome	24 (7)	34 (6)	0.78
Hepato-renal syndrome	2 (0.6)	2 (0.4)	0.84
Haemorragic fever	1 (0.3)	18 (3)	0.1
Unknown	17 (5)	12 (2)	0.24

^a^Khi-square test

^b^GFR < 60 mL/min/1.73m^2^

according to MDRD

### AKI etiologies

The etiologies are detailed in Table 1. Functional AKI was the main cause (46% (EC) vs. 39% (VC); *p* = 0.3): dehydration associated to drugs-induced renal hypoperfusion (i.e. renin-angiotensin-aldosterone blockers. Diuretics or non-steroidal anti-inflammatory drugs) accounted for 21% (EC) and 17% (VC) (*p* = 0.41). Acute tubular necrosis (ATN) represented 16% (EC) and 22% (VC) (*p* = 0.28) and glomerular disease 7% (EC) and 14% (VC) of etiologies (*p* = 0.09).

### Outcomes

The mean hospitalization durations were 12.6 ± 14.4 [1–143] (EC) and 12.9 ± 12.5 [1–91] (VC) days (*p* = 0.94) (Table 2). Dialysis requirement occurred in 24% (EC) and 25% (VC) of patients (*p* = 0.84). Renal recovery during in-hospital stay was more often complete in the EC compared to the VC cohort (65 vs. 45%; *p* = 0.004).

**Table 2:** Outcomes of the elaboration (EC) and validation (VC) cohorts

	EC (n = 323)		VC (n = 534)		
	n	%	n	%	p ^a^
Hospitalization duration^b^	12.6 [1–143]		12.9 [1–91]		0.94
Renal biopsy	38	12	76	14	0.59
Extrarenal depuration requirement	76	24	132	25	0.84
Death	50	16	48	9	0.15
sepsis	23	46	21	44	0.74
cardio-pulmonary failure	10	21	13	27	0.32
haemorrage	7	14	3	6	0.06
multiorgan failure	7	14	6	13	0.75
other causes of death	3	7	5	11	0.36
Complete renal recovery ^c^	179	65	219	45	0.004
Chronic dialysis	7	3	48	10	0.03
Destination					
home	149	46	307	57	0.21
other conventional unit	77	24	101	19	0.21
rehabilitation center	45	14	61	11	0.44
ICU	3	1	17	3	0.21

a Khi-square test

b days

c death-censored

Sixteen percent (EC) and 9% (VC) of patients died during their stay (*p* = 0.15). The main causes of in-hospital death were sepsis (46% (EC) vs. 43% (VC); *p* = 0.74), cardio-pulmonary failure (21% (EC) vs. 27% (VC); *p* = 0.35), and multiorgan failure (14% (EC) vs. 13% (VC); *p* = 0.32). After hospitalization, 46.1 (EC) and 57.4 (VC) % patients came back home, 23.8 (EC) and 18.9 (VC) % were secondarily transferred in another conventional medical unit. Only 0.8 (EC) and 3.3 (VC) % patients were admitted in an ICU, mainly for septic shock.

The comparison of dead and alive patients’ characteristics are summarized in the Additional file 1: Table S1.

### Elaboration and validation of the in-hospital death prognosis score

We analyzed all clinical and biological admission parameters significantly associated with in-hospital death in the EC. In multivariate analysis, multiple myeloma, low diastolic blood pressure, low Glasgow score, oxygen requirement, fluid overload, high phosphate levels, elevated white blood cell count, and low prothrombin time were significantly associated with in-hospital death (Table 3). We developed a score based on the ponderation coefficient of each associated parameters independently associated to in-hospital death from the multivariate logistic regression and obtained an equation named Probability of in-hospital death (P (id)) : (P (id) = 7.75–0.563 *Glasgow score - 0.025 * diastolic blood pressure + 2.26 * oxygen therapy + 0.68 * fluid overload + 0.00007 * white blood cell count + 1.021 * serum phosphate - 0.016 * prothrombin time + 1.72 (if presence of multiple myeloma)).

**Table 3:** In-hospital death associated clinical and biological parameters in univariate and multivariate logistic regression in the elaboration cohort

*Parameter*	Monovariate analysis	Multivariate analysis
Male gender	*p* = 0.859	
Age	*p* = 0.014	
CKD (GFR < 60 ml/min/1.73 m2)	*p* = 0.328	
Provenance	*p* = 0.517	
Multiple myeloma	*p* < 0.001	16.9 [2.1–140.7] *p* = 0.009
Systolic blood pressure	*p* < 0.001	
Diastolic blood pressure	*p* < 0.001	0.94 [0.88–0.98] *p* = 0.014
Glasgow score	*p* < 0.001	0.62 [0.40–0.95] *p* = 0.030
Urine output	*p* < 0.001	
Oxygen therapy	*p* < 0.001	8.6 [2.8–26.5] *p* < 0.001
Fluid overload	*p* < 0.001	2.87 [1.11–7.37] *p* = 0.030
urea	*p* = 0.656	
creatinine	*p* = 0.199	
sodium	*p* = 0.031	
potassium	*p* = 0.551	
bicarbonate	*p* = 0.729	
Adjusted calcemia	*p* = 0.011	
phosphoremia	*p* < 0.001	2.34 [1.24–4.42] *p* = 0.009
albuminemia	*p* = 0.001	
C-reactive protein	*p* = 0.113	
Hemoglobin	*p* = 0.413	
White blood cell count (/mm^3^)	*p* = 0.010	1.00 [1.00–1. 00] *p* = 0.018
Prothrombin time	*p* < 0.001	0.97 [0.94–0.99] *p* = 0.019
Thromboplastin time	*p* = 0.029	
Fibrinogen	*p* = 0.554	

The score applied to the VC resulted in a ROC curve with an AUC of 0.877 ± 0.0253 (*p* < 0.0001). The contingency tables comparing expected to observed death proportions in 10 patient groups randomly extracted from the VC did not show any good predictive power of the P (id) score and the Hosmer and Lemeshow test was not statistically significant (*p* < 0.0001) (data not shown). This suggested the absence of good calibration of the prognosis score equation derived from the EC applied to the VC.

**Fig. 1:**
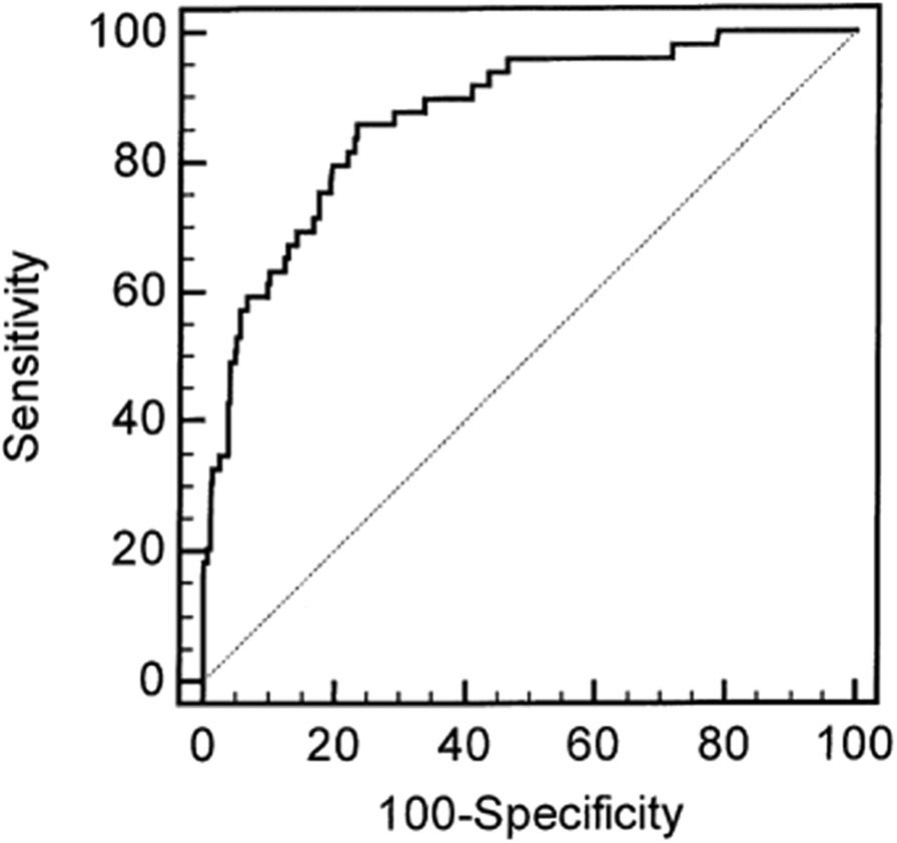
ROC curve of the in-hospital death prognosis score *P*_*2*_*(id)* applied to the elaboration cohort (EC): AUC 0.872 (*p* < 0.0001)

**Fig. 2:**
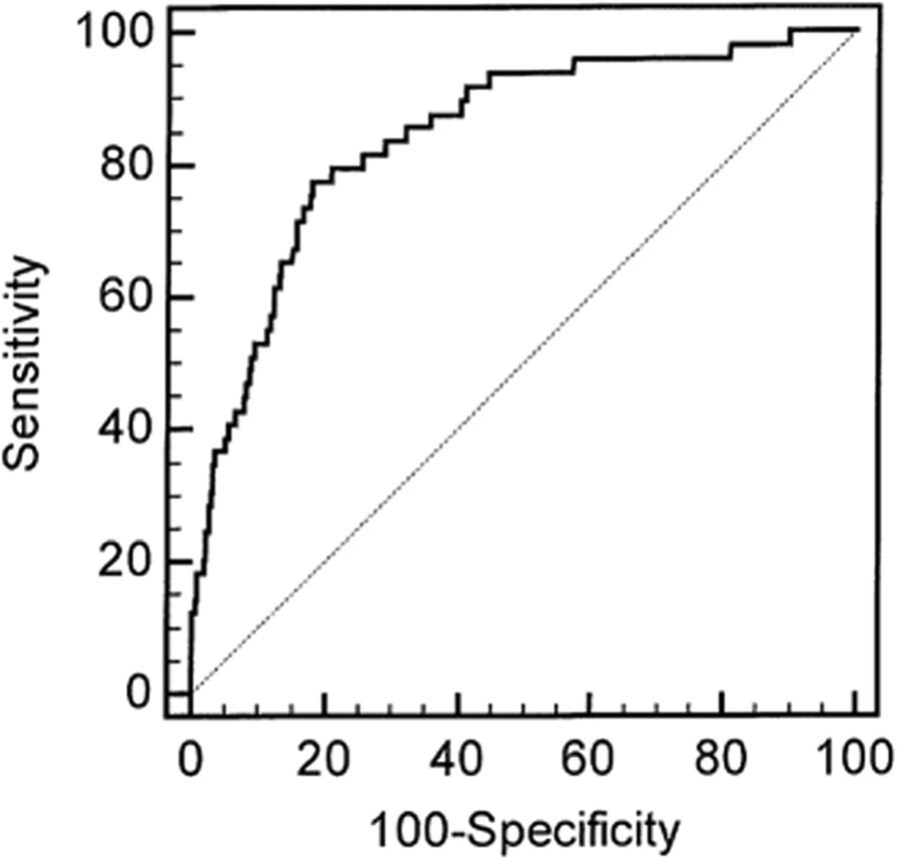
ROC curve of the in-hospital death prognosis score *P*_*2*_*(id)* applied to the validation cohort (VC): AUC 0.845 (*p* < 0.0001)

To improve the calibration of our score we compared admission parameters significantly associated with in-hospital death in both cohorts. The parameters independently associated with in-hospital death were comparable except for white blood cell count and serum phosphate which were not associated with in-hospital death in the VC. We therefore built a new EC-derived in-hospital death prognosis score named P_2_(id) after suppression of white blood cell count and serum phosphate (P2(id) = 7.75–0.563 *Glasgow score - 0.025 * diastolic blood pressure + 2.26 * oxygen therapy + 0.68 * fluid overload - 0.016 * prothrombin time + 1.72 (if presence of multiple myeloma)). We first applied the P_2_(id) score in the EC cohort: the AUC was 0.872 +/− 0.0275 (*p* < 0.0001) (Fig. 1). The Hosmer and Lemeshow test was statistically significant with a good adequation between expected and observed deaths (*p* = 0.94) (Additional file 2: Table S2). Thus, we applied the P_2_(id) score in the VC: the AUC was 0.845 +/− 0.0295 (p < 0.0001) (Fig. 2). The Hosmer and Lemeshow test was statistically significant with a good adequation between expected and observed deaths (*p* = 0.19) (Table 4).

**Table 4:** Contingency tables after application of the in-hospital death score P_2_(id) to the validation cohort using Hosmer and Lemeshow test

Group	Expected deaths	Observed deaths
1	1.694	1
2	1.745	1
3	1.737	0
4	1.779	1
5	1.838	0
6	1.955	4
7	2.393	3
8	4.067	6
9	7.883	12
10	23.908	21

Test Hesmer & Lemeshow *p* = 0.19

## Discussion

Most patients with AKI are admitted to conventional units. However, most studies have so far been conducted in the ICU setting [17–24], and therefore very few data exist on AKI in patients admitted to conventional medical units. Our study provides data on epidemiological aspects as well as etiology and outcome of AKI in a conventional unit of nephrology. Furthermore, our prospective study on AKI in two 4 year-period independent cohorts consisted in the elaboration of a score, based on simple available admission clinical and biological parameters, to stratify individual risk of in-hospital death and thus to evaluate the severity of the AKI episode at patient’s admission. In-hospital death was 15.5 and 8.9%, respectively in the EC and VC. Multiple myeloma, diastolic blood pressure, Glasgow score, oxygen therapy, fluid overload and prothrombin time were parameters at admission independently associated with the risk of in-hospital death. The prognosis death score derived from these parameters shows a good AUC in both cohorts. This is to our knowledge the first score established from patients admitted to a conventional nephrology unit for AKI.

Our data confirmed that AKI is a disease of the elderly; nearly half patients were 75 years-old or older. More than half patients were admitted from emergency room with many functional AKI. Consequently, comorbidities including cardiovascular disease, cancer and CKD were highly prevalent in this population, and this might also explain why drug nephrotoxicity was found to be the major cause of AKI. This underscores the need for a careful selection of potentially nephrotoxic medications in the elderly in addition to close monitoring and dose adaptation of drugs if relevant. Despite a median age over 75 years-old, our analysis did not reveal age as an independent risk factor for in-hospital death. Recent reports on AKI in conventional medical units also describe comparable proportions of old patients, much more than what is reported in ICU studies [12]. The elderly, so as the presence of cardiovascular or neoplastic comorbidities, could be one of the causes for contraindications of the patients’ admission in ICU. This could explain why old patients with AKI are more represented in cases admitted to conventional medical units. About 50% were CKD patients, highlighting renal diseases as a major risk factor for AKI as reported by others [25]. The secondary transfer from another conventional hospitalization unit in one third cases demonstrates the frequent iatrogenic origin of AKI. However iatrogenic AKI were not associated with poorer survival compared to community-acquired ones as reported by others [26–30].

The analysis of causes also showed high proportion of age-related AKI. Functional AKI remains the main cause, related to medicines such as NSAIDs, ACE inhibitors, ARA 2 or diuretics. Older age and polypharmacy are the cornerstone of functional AKI as reported by Anderson et al in a recent meta-analysis [31]. ATN were the second major cause, mainly due to prolonged ischemia or shock. Noteworthy, obstructive etiologies only represented 10% of cases in an old manly cohort, suggesting local recruitment bias: those patients are often first addressed to the urologist. In other studies, obstructive nephropathy and acute tubular necrosis also greatly contributed to AKI [14]. ATN, hepatorenal and cardiorenal syndromes, multiple myeloma were more observed in deceased compared to surviving patients, yet only multiple myeloma remained independently associated with death. Indeed, whatever the underlying mechanism, renal lesions are of bad prognosis during multiple myeloma. Moreover, those patients are more prone to sepsis because of their immunosuppressive status related to the disease itself or secondary to chemotherapy. Chertow et al showed immunosuppression as a major factor of death in ATN-associated AKI [32]. Selby and colleagues [26] described neoplasia as the third cause of death (after sepsis and cardiovascular events) during an AKI episode.

Renal replacement therapy was performed in 25% of AKI cases, and dialysis requirement was more frequent in deceased patients. Mesropian et al [33] reported only 3.6 and 5.1% of dialyzed patients, while Schissler et al [29] described 5.5 and 5.9%, respectively in community- and hospital-acquired AKI. On the contrary, 42 and 47% of cases were dialyzed in a Chinese report [30], yet including ICU patients. This shed light on the extreme subjective criteria for dialysis initiation, probably depending on the physician and the comorbidities and age of patients. Hsu et al demonstrated that older patients with high number of comorbidities are less prone to benefit from invasive procedures accompanying renal replacement therapy [6]. Interestingly, a recent study showed a lower in-hospital mortality when physicians adhere to an algorithm providing recommendations on RRT initiation, yet only for low disease severity cases [34].

Mortality rate for AKI patients in ICU is reported to be as high as 50% in most studies, likely a reflection of multiple comorbidities [9]. We observed a significantly lower mortality rate in our patients, which was expected. Nevertheless, AKI remains a potentially fatal disease, even in patients not requiring admission to ICU, with an overall mortality rate of about 10%, mainly from sepsis and cardiopulmonary failure when pooling our 2 cohorts. Two recent studies reported 10.8% [35] and 21.9% [26] death with comparable proportions of death causes. Our study confirmed that, in addition to certain causes of AKI, coexistence of non-renal organ failure is a powerful predictor of death in patients with AKI: diastolic blood pressure better reflects mean arterial pressure [36] and hypotension (systolic blood pressure) was earlier integrated to SHARF prognosis score for AKI [37], helping physicians to take decision about patient admission in ICU; low Glasgow score is significantly associated with death in our study confirming what was earlier reported by Liano et al [38]; oxygen requirement is often integrated to AKI prognosis scores developed in ICU, where requirement to artificial ventilation is needed in cardiac or pulmonary failures and systematically associated with death [19, 32, 37–40], yet it could also reflect fluid overload, parameter we also reported as an independent risk factor for death in our study and integrated to our score. Indeed, several reports in ICU demonstrated that a fluid overload defined as a 10% increase in body weight was associated with death and non-renal recovery after AKI [41, 42]. A recent meta-analysis reported positive fluid balance as a parameter associated with multiorgan failure (i.e. brain, cardiac, pulmonary, hepatic, digestive, renal and skin) and suggested systematic early requirement to renal replacement therapy when diuretics are not efficient to control fluid overload [43].

There are limitations to our study. Firstly, in patients with previous renal impairment, the definition of AKI as an acute increase of 100 μmol/L could seem arbitrary and introduce a selection bias in our study. In fact, the inclusion of patients in the first cohort (EC) started in 2001. There was no clear universal laboratory definition. No real value of acute increase in serum creatinine was available for patients with previous renal impairment. Hence, we chose to arbitrary double the creatinine value increase in patients with previous kidney impairment as compared with what was defined for patients with previous normal renal function in KDIGO. Later, Singri et al. in 2003 [44] and Lameire et al. in 2005 [45] defined acute kidney impairment as an acute and durable increase in serum creatinine of 44.2 μmol/l, if basal serum creatinine was below 221 μmol/l or an increase in serum creatinine above 20% of the basal creatinine, if its value was above 221 μmol/l. To keep the prospective design of our study and to respect the same design for the VC, we did not change the initial definition for patients with previous renal impairment. Nevertheless patients who were admitted to our ward for AKI had rarely an increase in serum creatinine below 100 μmol/L. Thus, we should have missed little number of AKI cases, as the other cases were often managed as ambulatory patients. Secondly, this is a single center epidemiological study and it is then possible that local circumstances have affected the spectrum of etiologies of AKI found in this population. In fact, our university hospital is considered a tertiary center, and some of the patients included in this study have been transferred from smaller regional medical centers for different reasons (e.g. failure of preliminary treatment or need for plasma exchange). Therefore, severity of AKI as well as outcome and mortality might have been affected. Thirdly, in most cases, the etiology of AKI has been determined based on clinical picture and laboratory data without histologic confirmation. However, it can be considered the routine practice and standard of care in a conventional unit where all patients do not undergo a kidney biopsy.

Our prognosis score was built after completion of all inclusions on two prospective and time-independent cohorts with few missing data and thus was free from recruitment or intervention biases. Our primary outcome was death and could not suffer from judgment bias. We strictly respected the policy for the elaboration and validation steps to build our score. Nevertheless, we had to adjust the equation of our score derived from the elaboration cohort because of an imperfect match between expected (calculated by the score) and observed events after application of the equation in the validation cohort. Both cohorts were not strictly comparable as recruitment has changed between the 2 periods. Etiologies of AKI were differently represented for functional AKI related to medications (1.5 vs 10.5%; *p* = 0.007, respectively in the VC and the EC) and ATN related to medications toxicity (5.8 vs 0.3%; *p* = 0.03, respectively in the VC and the EC). These differences probably explain why total renal recovery was significantly lower and permanent renal death significantly higher in the VC. Moreover serum phosphate and white blood cell count were different between both cohorts. Serum phosphate ranges from 0.68 to 5.35 (mean value: 1.79 ± 0.66 mmol/L, median 1.65 mmol/L) in the EC and from 0.35 to 2.74 mmol/L (mean value 1.76 ± 0.59 mmol/L, median 1.74) in the VC. WBC count ranges from 2000 to 34,400/mm^3^ (mean value: 10,175 ± 5145/mm^3^, median 8800/mm^3^) in the EC and from 600 to 79,000/mm^3^ (mean value 10,401 ± 9000/mm^3^, median 6352/mm^3^) in the VC. Thus these two variables had to be removed in order to agree in both populations.

Although, the first score built in the elaboration cohort was predictive of death in both cohorts, its discriminative power was low. Suppression of two variables improved discrimination ability. This pragmatic approach is based on feedback from observation to improve the model. We finally compared independent death-associated factors of each cohort to only retain 7/9 common factors to both cohorts. Thus, the second equation was built from the elaboration cohort and validated in both cohorts with a good prediction of events in the validation cohort. Our score is highly specific but weakly sensitive, with decreased proportion of false positive cases. This could potentially help clinicians to better discriminate between high and low risk patients of in-hospital death.

Yet, if we here demonstrated the time validation of our score, we could not affirm its complete validation, due to the monocentric design of our study. A further validation in an independent conventional nephrology unit is needed now before its use in clinical practice. Furthermore, the capability of our score to discriminate between patients, either as a clinical decision tool for ICU admission, or for renal replacement therapy initiation is not demonstrated and should be used in future studies to demonstrate its clinical relevance.

## Conclusion

Our in-hospital death prognosis score is the first to be prospectively developed and validated for AKI admitted in conventional care units. Based on current parameters, easily collected at time of admission, this score could be a useful tool for physicians and nephrologists to determine the in-hospital death prognosis of this AKI population.

## Supplementary information

**Additional file 1: Table S1.** Comparison between dead and alive patients pooling patients from elaboration and validation cohorts. (DOCX 15 kb)

**Additional file 2: Table S2.** Contingency tables after application of the in-hospital death score P_2_(id) to the elaboration cohort using Hosmer and Lemeshow test. (DOCX 12 kb)

## Data Availability

The datasets used and/or analysed during the current study are available from the corresponding author on reasonable request.

## Abbreviations

AKI: Acute kidney injury
ATN: Acute tubular necrosis
CKD: chronic kidney disease
CRP: C-reactive protein
EC: elaboration cohort
GFR: glomerular filtration rate
ICU: intensive care units
MDRD: Modification of the Diet in Renal Disease
P (id): Probability of in-hospital death
SCr: serum creatinine
VC: validation cohort
