# Supplementary material for: Acute kidney injury in non-critical care setting: elaboration and validation of an in-hospital death prognosis score
Source: BMC Nephrol. 2019 Nov 21;20:419. doi: 10.1186/s12882-019-1610-9 (PMC6868787; doi:10.1186/s12882-019-1610-9)
Supplement: Supplementary file 1 — Additional file 1: Table S1. Comparison between dead and alive patients pooling patients from elaboration and validation cohorts. (DOCX 15 kb) [file 12882_2019_1610_MOESM1_ESM.docx]

| **Table S1: Comparison between dead and alive patients pooling patients from elaboration and validation cohorts.** | | |  |  |  |  |
| --- | --- | --- | --- | --- | --- | --- |
|  | **Deceased patients (n = 98)** | |  | **Surviving patients (n = 759)** | |  |
|  | **n** | **%** |  | **n** | **%** | **p ^1^** |
| Male gender | 60 | 61.2 |  | 468 | 61.6 | 0.95 |
| Mean age [min-max] | 72.2 [50 - 96] |  |  | 70.8 [18 - 100] | | 0.82 |
| CKD^2^ | 52 | 54 |  | 389 | 51.3 | 0.7 |
| Provenance |  |  |  |  |  |  |
| Home | 6 | 6.1 |  | 86 | 11.3 | 0.19 |
| Emergency | 50 | 51 |  | 410 | 54 | 0.67 |
| Other hospital unit | 40 | 40 |  | 242 | 31.7 | 0.22 |
| Rehabilitation center | 3 | 2.9 |  | 21 | 3 | 0.96 |
| Etiologies |  |  |  |  |  |  |
| Functional | 18 | 18.5 |  | 339 | 45.9 | 0.0003 |
| Dehydratation | 6 | 6.1 |  | 150 | 19.7 | 0.004 |
| Drugs | 2 | 2.3 |  | 40 | 5.3 | 0.26 |
| Both | 10 | 10.1 |  | 149 | 20.9 | 0.03 |
| Obstructive | 3 | 3 |  | 63 | 8.1 | 0.12 |
| Glomerular disease | 5 | 5 |  | 90 | 11.6 | 0.09 |
| ATN | 40 | 40.8 |  | 127 | 16.7 | 0.0001 |
| Post-ischemia | 30 | 30.6 |  | 80 | 10.5 | 0.0004 |
| Nephrotoxic agents | 4 | 4.1 |  | 28 | 3.6 | 0.85 |
| Rhabdomyolysis | 6 | 6.1 |  | 19 | 2.6 | 0.22 |
| Vascular disease | - | - |  | 28 | 3.5 | 0.059 |
| Multiple myeloma | 9 | 9.2 |  | 24 | 3.1 | 0.04 |
| Cardiorenal syndrome | 15 | 15.3 |  | 43 | 5.4 | 0.02 |
| Hepatorenal syndrome | 4 | 4.1 |  | 0 | 0 | 0.04 |
| Haemorragic fever | 0 | 0 |  | 19 | 2.5 | 0.11 |
| Unknown | 4 | 4.1 |  | 26 | 3.2 | 0.9 |
| Hospitalisation duration (days) | 15.5 [ 1 - 91 ] |  |  | 12.4 [ 1 - 143 ] | | 0.002 |
| Dialysis | 49 | 50 |  | 161 | 21.2 | 0.0002 |
| Renal biopsy | 7 | 7.2 |  | 107 | 14 | 0.11 |
| ^1^ : Khi-square test |  |  |  |  |  |  |
| ^2^ : GFR < 60 mL/min/1.73m^2^ according to MDRD | |  |  |  |  |  |
