# Supplementary material for: Acute kidney injury in non-critical care setting: elaboration and validation of an in-hospital death prognosis score
Source: BMC Nephrol. 2019 Nov 21;20:419. doi: 10.1186/s12882-019-1610-9 (PMC6868787; doi:10.1186/s12882-019-1610-9)
Supplement: Supplementary file 2 — Additional file 2: Table S2. Contingency tables after application of the in-hospital death score P2(id) to the elaboration cohort using Hosmer and Lemeshow test. (DOCX 12 kb) [file 12882_2019_1610_MOESM2_ESM.docx]

**Table S2:** **Contingency tables after application of the in-hospital death score P_2_(id) to the elaboration cohort using Hosmer and Lemeshow test.**

| **Group** | **Expected deaths** | **Observed deaths** |
| --- | --- | --- |
| 1 | 0.365 | 0 |
| 2 | 0.549 | 1 |
| 3 | 0.683 | 1 |
| 4 | 0.883 | 0 |
| 5 | 1.485 | 2 |
| 6 | 2.214 | 2 |
| 7 | 4.285 | 4 |
| 8 | 7.283 | 8 |
| 9 | 11.52 | 13 |
| 10 | 19.732 | 18 |
| Test Hesmer & Lemeshow p = 0.94 | |  |
